# Quantum Computing for Polymeric Composites: Principles, Methods, Applications, and Future Perspectives

**DOI:** 10.3390/polym18192418

**Published:** 2026-10-03

**Authors:** Min Ook Kim

**Affiliations:** Department of Civil Engineering, Seoul National University of Science and Technology, 232 Gongneung-ro, Nowon-gu, Seoul 01811, Republic of Korea; minookkim@seoultech.ac.kr

**Keywords:** quantum computing, polymeric composites, quantum algorithms, quantum machine learning, density functional theory, molecular dynamics, multiscale modelling, materials informatics, hybrid quantum–classical computing, materials design

## Abstract

Polymeric composites have become indispensable engineering materials owing to their exceptional mechanical performance, lightweight characteristics, corrosion resistance, and design flexibility. Conventional computational approaches, including density functional theory (DFT), molecular dynamics (MD), finite element analysis (FEA), and machine learning (ML), have substantially improved the understanding of polymer structure–property relationships. However, the increasing complexity of multiscale polymeric systems, intricate fiber–matrix interactions, and the computational cost associated with accurately modelling quantum-mechanical phenomena continue to present significant challenges for classical computing. This review provides a comprehensive assessment of the emerging role of quantum computing in polymeric composite research. It first introduces the fundamental principles of quantum computing, including qubits, quantum gates, quantum circuits, representative quantum algorithms, and contemporary hardware platforms. Subsequently, the review examines conventional computational methods used for modelling polymeric composites and discusses how quantum computing may complement and accelerate these established approaches. Recent and prospective applications in polymer electronic structure calculations, fiber–matrix interface engineering, nanocomposite design, multiscale modelling, and intelligent materials discovery are critically evaluated. Particular emphasis is placed on hybrid quantum–classical frameworks that integrate quantum computing with artificial intelligence, materials informatics, and high-throughput computational workflows as candidate tools for polymer design. The evidence is separated into polymer-specific hardware demonstrations, simulator-based studies, transferable non-polymer benchmarks, and prospective applications. Existing studies establish feasibility for selected tasks; they do not establish general end-to-end quantum advantage for realistic polymeric composites.

## 1. Introduction

Polymeric composites have become one of the most important classes of advanced engineering materials because of their high specific strength, lightweight characteristics, excellent corrosion resistance, superior fatigue performance, and exceptional design flexibility. By combining polymer matrices with reinforcing fibers, nanoparticles, or other functional fillers, these materials can be tailored to achieve a wide range of mechanical, thermal, electrical, and chemical properties. Consequently, polymeric composites have found widespread applications in aerospace, automotive, civil infrastructure, marine engineering, biomedical devices, electronics, and renewable energy systems, where the demand for lightweight, durable, and multifunctional materials continues to increase [1,2,3,4]. The rapid development of advanced reinforcements, including carbon nanotubes (CNTs), graphene, MXenes, and other two-dimensional nanomaterials, has further expanded the performance envelope of polymeric composites, supporting improvements in mechanical, thermal, and electrical properties [2,3,4,5,6].

The outstanding performance of polymeric composites arises from complex interactions occurring across multiple spatial and temporal scales. Their macroscopic behaviour is governed not only by the intrinsic properties of the constituent materials but also by molecular architecture, polymer chain dynamics, reinforcement morphology, fiber orientation [7], interfacial adhesion, processing conditions, and microstructural defects. These hierarchical interactions influence fundamental properties such as stiffness, strength, fracture toughness, thermal conductivity, electrical conductivity, and long-term durability. Understanding the relationships between processing, microstructure, and performance therefore remains one of the central challenges in polymer science and composite engineering [1,3,4]. Over the past several decades, computational materials science has become an indispensable tool for investigating these multiscale phenomena. Density functional theory (DFT) has enabled accurate predictions of electronic structures, chemical bonding, and reaction mechanisms at the atomic scale, whereas molecular dynamics (MD) simulations have provided detailed insights into polymer chain mobility, cross-linking behaviour, interfacial interactions, and nanoscale deformation mechanisms. At larger scales, finite element analysis (FEA), continuum mechanics, and multiscale modelling have been widely employed to evaluate stress transfer, fracture behaviour, damage evolution, and structural integrity. More recently, artificial intelligence (AI), machine learning (ML), and materials informatics have accelerated materials discovery by enabling data-driven prediction of material properties, optimization of processing parameters, and inverse materials design [8,9,10,11].

Despite these advances, significant computational challenges remain. Highly accurate treatments of electronic correlation can become expensive as the number of electrons and orbitals increases, motivating quantum algorithms for selected electronic-structure subproblems [12]. This difficulty should be distinguished from the cost of large classical MD models or multiscale mechanical simulations. Moreover, near-term quantum processors introduce their own noise, coherence and circuit-depth limitations, so a theoretical algorithmic advantage does not establish a practical advantage for polymeric composites [13].

Recent developments in quantum computing offer a fundamentally different computational paradigm capable of addressing several of these limitations. Unlike conventional digital computers that process information using binary bits, quantum computers manipulate quantum bits (qubits), which exploit the principles of superposition, entanglement, and quantum interference to encode and process information. These uniquely quantum-mechanical properties enable certain computational problems to be solved more efficiently than the best-known classical approaches for certain tasks. Since Feynman’s pioneering proposal that quantum systems could be efficiently simulated by quantum computers [14], followed by Deutsch’s formulation of the universal quantum computer [15], quantum computing has evolved into one of the most rapidly developing interdisciplinary research fields. Landmark algorithms developed by Shor [16] and Grover [17] demonstrated that quantum computers possess computational advantages for specific classes of problems, stimulating intensive research into quantum algorithms, quantum hardware, and quantum software.

The emergence of noisy intermediate-scale quantum (NISQ) devices has further accelerated interest in practical quantum computing for scientific and engineering applications. Although current quantum processors remain constrained by limited qubit numbers, decoherence, and hardware noise, hybrid quantum–classical algorithms have demonstrated promising capabilities in quantum chemistry, molecular simulations, optimization, and machine learning. Variational Quantum Eigensolvers (VQE), Quantum Approximate Optimization Algorithms (QAOA), and quantum machine learning (QML) techniques have attracted particular attention because they are compatible with existing NISQ hardware while providing potential computational advantages for selected problems in materials science [18,19,20,21].

Within polymeric composite research, quantum computing presents a particularly promising opportunity. Many of the challenges associated with polymer design—including prediction of electronic structures, polymerization mechanisms, cross-linking reactions, fiber–matrix interfacial bonding, molecular adsorption, and nanoscale reinforcement behaviour—are fundamentally quantum-mechanical in nature. Quantum algorithms may therefore provide improved accuracy and computational efficiency for these problems compared with conventional approaches. Furthermore, integrating quantum computing with established computational techniques such as DFT, MD, FEA, and AI-based materials informatics may establish hybrid computational frameworks capable of accelerating the discovery and optimization of advanced polymeric composites. Such frameworks could facilitate inverse materials design, high-throughput virtual screening, and autonomous materials discovery while reducing development time and computational cost.

Although the application of quantum computing to chemistry, condensed matter physics, and quantum materials has expanded rapidly during the past decade, its application to polymeric composites remains relatively unexplored. Existing studies are dispersed across multiple disciplines, including quantum information science, computational chemistry, polymer physics, materials engineering, and computer science. Consequently, there remains a lack of comprehensive reviews that critically synthesize these interdisciplinary developments while evaluating their relevance to polymeric composite research. Moreover, significant scientific and technological challenges—including quantum hardware limitations, algorithm scalability, software interoperability, benchmark datasets, and validation against experimental observations—must be addressed before quantum computing can become a routine tool for composite materials engineering.

Existing reviews mainly address quantum chemistry or quantum-enabled materials design at a general level [12,22,23,24], whereas polymeric composites introduce distinct questions associated with chain conformation, cross-linked networks, heterogeneous interfaces, and multiscale transfer to engineering performance. The specific contribution of this review is therefore to connect these polymer-specific problems with demonstrated quantum algorithms and hybrid DFT/MD/FEA/AI workflows, while distinguishing experimentally or computationally demonstrated cases from prospective applications and identifying the validation requirements that remain before practical deployment.

**Figure 1:**
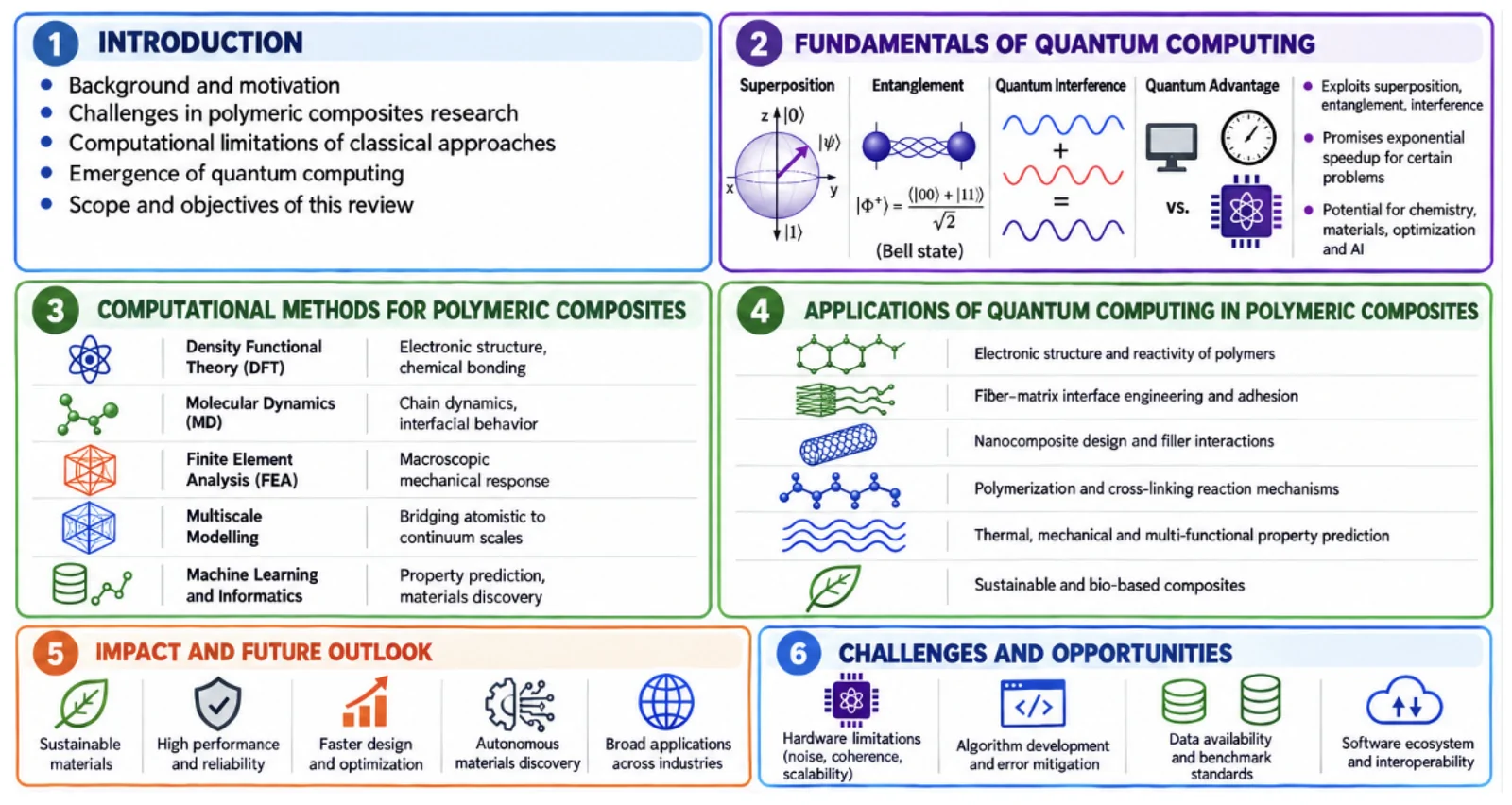
Conceptual framework of quantum computing for polymeric composites: from computational foundations to next-generation materials design.

Accordingly, this review aims to provide a comprehensive and critical assessment of the emerging role of quantum computing in polymeric composite research. First, the fundamental principles of quantum computing, including quantum information processing, representative quantum algorithms, and contemporary hardware architectures, are introduced. Subsequently, conventional computational approaches used in polymeric composite modelling are reviewed to establish the current state of the art before discussing how quantum computing can complement and accelerate these methodologies. Emerging applications in polymer molecular modelling, fiber–matrix interface engineering, nanocomposite design, multiscale simulations, and intelligent materials discovery are then critically examined. Finally, the review discusses current limitations, identifies key research gaps, and outlines future research directions for integrating quantum computing with artificial intelligence, materials informatics, and advanced computational materials science. By consolidating recent advances from quantum information science and polymer engineering, this review aims to provide researchers with a comprehensive framework for understanding the opportunities, challenges, and future prospects of quantum computing in the development of next-generation high-performance polymeric composites. Figure 1 presents the overall conceptual framework of this review.

## 2. Fundamentals of Quantum Computing

### 2.1. Historical Development of Quantum Computing

Quantum computing uses quantum states and operations to process information. Unlike a classical bit, a qubit can occupy a coherent superposition of its two computational-basis states. Quantum algorithms offer advantages for certain well-defined tasks, while potential benefits in chemistry, optimization and machine learning depend on the problem, algorithm and implementation [12,13,14,15,16,17,18,19,20,21]. These principles motivate testing quantum processors as specialized components of classical materials workflows.

The historical development of quantum computing has been reviewed extensively elsewhere [14,15,16,17,25]. For the present polymer-focused review, only milestones directly relevant to computational materials science are retained: quantum simulation, variational algorithms, error mitigation, and hybrid quantum–classical execution. Shor’s and Grover’s algorithms established important examples of quantum speedup, but their detailed derivations are outside the scope of this review [16,17].

Over the past decade, remarkable progress has been achieved in quantum hardware. Superconducting circuits, trapped-ion processors, neutral-atom systems, and photonic quantum computers have evolved from laboratory prototypes to programmable quantum devices capable of executing increasingly complex algorithms. Although current systems remain constrained by limited qubit numbers, hardware noise, and decoherence, they have initiated the NISQ era, in which hybrid quantum–classical algorithms are being actively explored for practical scientific applications [13,20,21].

For computational materials science, these developments are particularly important because atoms, molecules, and condensed-matter systems inherently obey quantum mechanics. Consequently, quantum computing provides a natural computational framework for investigating electronic structures, molecular interactions, and chemical reactions that are difficult to describe accurately using classical computational approaches. Whether quantum computing becomes a useful complement to conventional methods in polymeric composite research will depend on validated accuracy, resource requirements, and end-to-end cost for specific tasks.

### 2.2. Fundamental Principles of Quantum Computing

The computational capability of quantum computers arises from four fundamental principles of quantum mechanics: superposition, entanglement, quantum interference, and quantum measurement. Together, these principles distinguish quantum information processing from classical computation and provide the theoretical basis for quantum algorithms. Among these principles, quantum superposition is perhaps the most fundamental. Whereas a classical bit exists exclusively in one of two logical states (0 or 1), a qubit may exist as a coherent linear combination of both basis states,

(1) |ψ⟩=α|0⟩+β|1⟩

where *α* and *β* are complex probability amplitudes satisfying the normalization condition

(2) $${\left|\alpha \right|}^{2}+{\left|\beta \right|}^{2}=1$$

An n-qubit register spans a Hilbert space of dimension 2^n^, which permits a compact representation of quantum amplitudes. This exponential growth of state-space dimension should not be equated with exponential computational speedup: any advantage is algorithm- and problem-dependent. For computational chemistry and materials science, the key relevance is the ability to represent correlated molecular wavefunctions and electronic configurations in a form natural to quantum hardware. A second key principle is quantum entanglement, which describes joint quantum states that cannot be expressed as mixtures of product states of the individual qubits. Entanglement can encode electron correlation in molecular systems and is therefore important for quantum simulations of chemically active regions in polymers and composite interfaces. For the present review, quantum interference is considered mainly through its practical effect on variational-circuit design, sampling efficiency, and the dependence of measured expectations on coherent quantum evolution in VQE, QAOA, and QML workflows [18,19,20,21]. Likewise, measurement is treated here as a practical source of sampling cost and uncertainty in quantum materials calculations; detailed gate-level and measurement formalism can be found in standard references [22,25].

Although these principles differ fundamentally from classical information processing, they should not be regarded merely as theoretical concepts. Their practical importance lies in their ability to improve simulations of molecular and electronic systems that underpin computational materials science. For polymeric composites, quantum mechanical descriptions of polymer chains, fiber–matrix interfaces, nanoparticle functionalization, and cross-linking reactions are expected to benefit from these computational capabilities as quantum hardware continues to evolve. Accordingly, quantum computing should be viewed as a complementary technology that extends, rather than replaces, established computational techniques such as density functional theory, molecular dynamics, and finite element analysis.

### 2.3. Quantum Information Processing and Hardware

Building on the qubit concepts introduced in Section 2.2, this subsection focuses on physical implementation and circuit operations rather than redefining superposition and entanglement. For n qubits, the underlying Hilbert-space dimension is 2^n^; this is a representational property, not a statement that computational capacity or speedup grows exponentially for generic problems.

Physical approaches to qubits include superconducting circuits and trapped ions, among other platforms [22,25,26,27,28]. They differ in coherence, gate speed, fidelity, connectivity and control requirements. Superconducting processors and trapped-ion systems provide established examples with distinct engineering trade-offs [26,27,28]. Their suitability for a polymer calculation depends on the algorithm and required resources.

Quantum computation is performed through quantum gates, which are unitary operations that manipulate quantum states while preserving coherence. Single-qubit gates, including the Pauli-X, Pauli-Y, Pauli-Z, Hadamard, and phase gates, modify individual qubits, whereas multi-qubit gates, particularly the controlled-NOT (CNOT) gate, generate entanglement between qubits. By combining these elementary operations into quantum circuits, complex computational tasks can be executed through coherent quantum evolution.

Although today’s quantum processors remain limited by hardware noise and decoherence, continuous improvements in qubit fidelity and quantum error mitigation are steadily enhancing computational performance. These developments provide the technological foundation for applying quantum information processing to computational chemistry, molecular simulations, and materials design.

### 2.4. Quantum Algorithms for Materials Science

The practical value of quantum computing is determined not only by hardware but also by the development of efficient quantum algorithms. Several algorithms have demonstrated considerable potential for computational chemistry and materials science, particularly in solving problems involving electronic structure calculations, optimization, and data-driven materials discovery. Among these, the VQE has emerged as one of the most promising algorithms for NISQ devices. VQE combines quantum state preparation with classical optimization to estimate molecular ground-state energies while maintaining relatively shallow quantum circuits [18,20]. Consequently, VQE has become a leading approach for quantum chemistry simulations involving molecules and functional materials. Another important algorithm is Quantum Phase Estimation (QPE), which provides highly accurate eigenvalue calculations for quantum systems. High-precision QPE for chemically demanding systems is expected to require fault-tolerant resources beyond present NISQ demonstrations [12,22]. QAOA and Ising/QUBO formulations provide frameworks for combinatorial optimization [29,30,31]. Their application to topology, processing, reinforcement distribution and inverse polymer-composite design is prospective and requires explicit problem formulations and benchmarks. In parallel, QML explores quantum approaches to learning tasks, including classification and regression [19]. Hybrid quantum–classical learning frameworks are increasingly being explored for materials informatics because they combine the strengths of conventional machine learning with quantum models whose benefit must be established for each task. Although practical demonstrations remain limited, the contribution of QML to materials discovery remains to be demonstrated through application-specific comparisons. For polymeric composites, accelerated discovery through QML remains a hypothesis to be tested against matched classical learning models, including data-loading, training, and measurement costs. Collectively, these algorithms demonstrate that quantum computing is not intended to replace established computational techniques but rather to complement them by addressing computational bottlenecks associated with high-dimensional optimization and quantum-mechanical simulations.

### 2.5. Quantum Computing for Computational Materials Science

**Figure 2:**
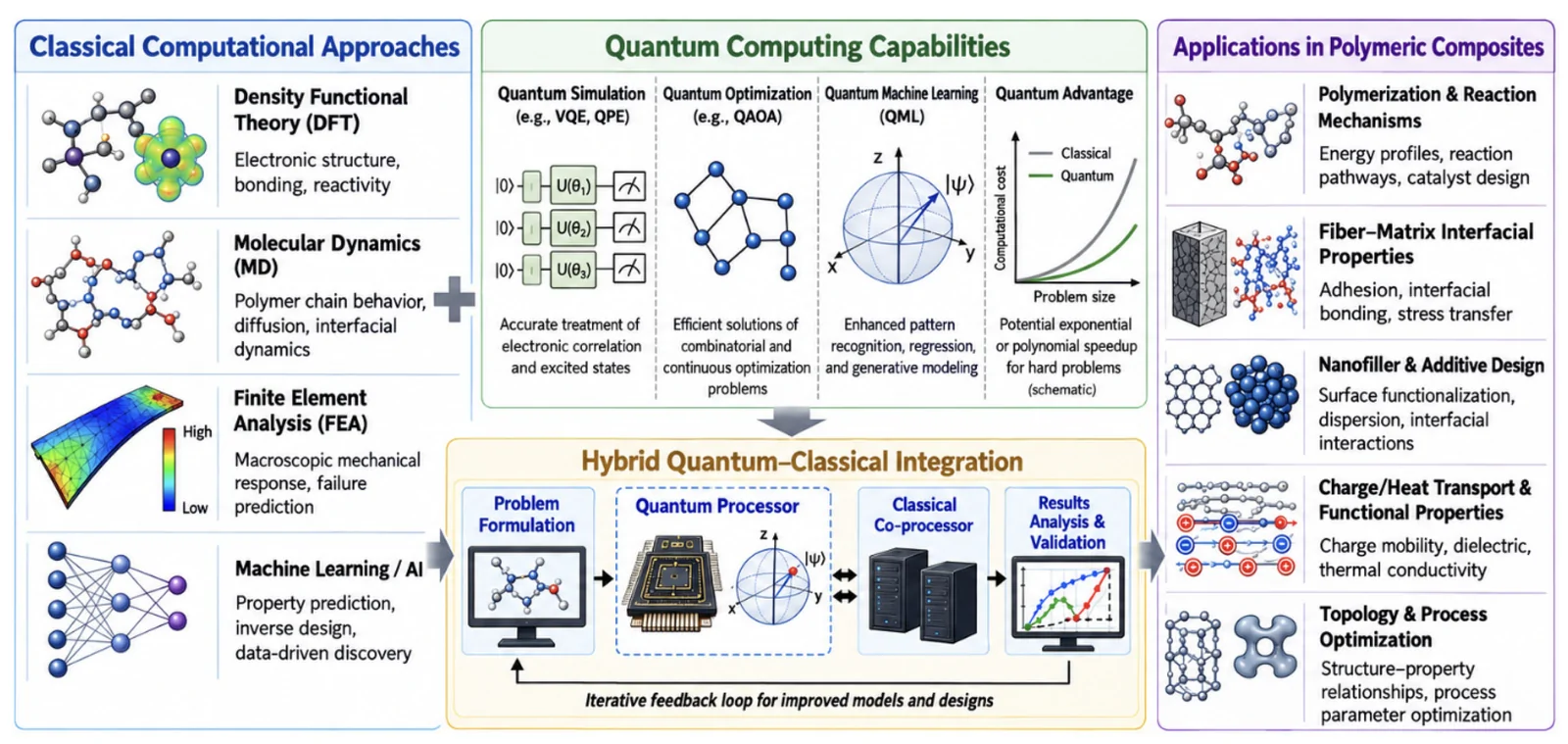
Hybrid quantum–classical computational framework for polymeric composite research.

Recent progress in quantum computing for computational materials science is better described through benchmarked hybrid workflows than through a generic expectation that quantum processors will replace DFT, MD, or FEA. Battaglia et al. [32] implemented a periodic active-space embedding framework that couples CP2K with Qiskit Nature and applies the VQE and quantum equation-of-motion algorithms; the framework was demonstrated for localized defect states in MgO and illustrates how a quantum solver can be embedded within a mature electronic-structure code. Pollard and Choudhary [33] introduced BenchQC and systematically benchmarked VQE within a quantum–DFT embedding workflow for small aluminium clusters by varying circuit ansätze, optimizers, basis sets, simulators, and noise models, thereby providing a reproducible assessment of accuracy and computational sensitivity. More recently, Barroca et al. [34] applied sample-based quantum diagonalization and its extended variant to an oxygen-reduction surface reaction, mapping the largest circuits to 80 qubits of an IBM Heron R2 processor and benchmarking tractable active spaces against classical configuration-interaction calculations. Polymer-specific demonstrations remain fewer but are no longer entirely prospective. Micheletti et al. [35] reformulated lattice-polymer statistical mechanics as a quadratic discrete optimization problem and sampled dense polymer mixtures on a D-Wave quantum annealer. For engineering composites, Cho and Cheon [36] applied quantum annealing to optimize Cu-film placement in CFRP for through-thickness thermal conductivity, while Cho et al. [37] used a QUBO-based quantum-annealing formulation for natural-fibre hybrid laminate stacking and impact-performance design. Recent analysis of quantum chemistry beyond ground-state calculations further emphasizes that useful scientific capability depends jointly on algorithmic development and fault-tolerant hardware rather than on physical-qubit count alone [38]. These concrete benchmarks and implementations indicate that a defensible near-term research target is the evaluation of quantum computing as a hybrid solver for selected correlated-electron, sampling, or discrete-optimization subproblems, with DFT, MD, FEA, AI, and HPC remaining responsible for the surrounding multiscale workflow. Accordingly, performance should be evaluated using end-to-end accuracy, scaling, logical-qubit resources, circuit depth, measurement and optimization overhead, and wall-clock cost, rather than by the nominal number of physical qubits alone. Figure 2 summarizes this hybrid quantum–classical perspective.

In the hybrid workflow in Figure 2, classical simulation or experiment defines the polymer-specific subproblem and supplies descriptors, Hamiltonian terms, or design constraints. The selected electronic-structure, sampling, or QUBO subproblem is encoded for the quantum solver, and measured outputs return to a classical optimizer or analysis routine. Only validated results are transferred to MD, FEA, materials informatics, or experimental evaluation.

## 3. Computational Methods for Polymeric Composite Modelling

### 3.1. Overview

Computational modelling has become an indispensable tool for understanding, designing, and optimizing polymeric composites. Owing to their hierarchical architectures and complex physicochemical interactions, polymeric composites exhibit material behaviour across multiple spatial and temporal scales, ranging from atomic-level chemical bonding to structural-scale mechanical performance. Consequently, no single computational methodology can accurately describe all relevant physical phenomena, and complementary modelling techniques are generally integrated within multiscale computational frameworks to provide comprehensive predictions of material behaviour and performance [39,40,41].

**Figure 3:**
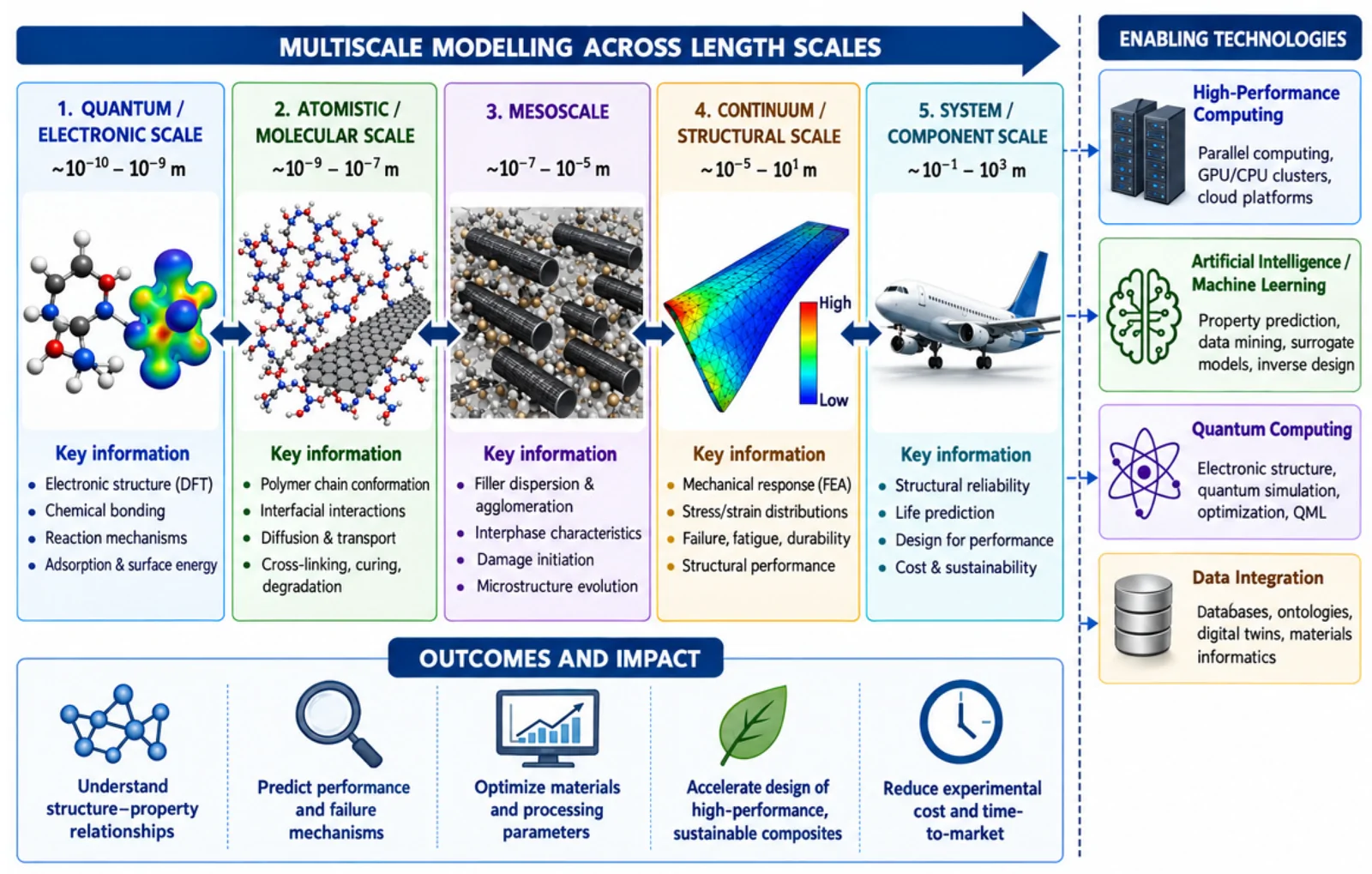
Conceptual overview of multiscale computational modelling for polymeric composites.

To keep the review focused on quantum computing, Section 3.2, Section 3.3, Section 3.4 and Section 3.5 are intentionally restricted to the quantum-relevant bottlenecks of established methods—electronic-structure cost, force-field transferability, multiscale parameter transfer, high-dimensional optimization, and data limitations. Standard derivations and broad application surveys available in specialist reviews are omitted; Figure 3 and Table 1 provide only the cross-scale context needed for Section 4 [39,40,41].

### 3.2. Density Functional Theory

DFT is treated here as the electronic-structure baseline against which quantum-assisted methods are assessed; emphasis is placed on polymer-specific limitations and quantum–classical coupling rather than on the formal derivation of DFT [42,43,44].

DFT uses the electron density to determine ground-state properties within a chosen exchange–correlation approximation [42,43,44]. It provides a classical electronic-structure baseline for local bonding, adsorption and reaction calculations, but its accuracy depends on the functional, dispersion treatment, structural model and relevant electronic correlations. Application-specific evidence is therefore needed in addition to foundational DFT references. Table 2 identifies primary studies of graphene/graphene oxide–epoxy adhesion [45], CNT–epoxy interfaces [46], MXene–epoxy binding [6], and epoxy–amine curing [47]. These are classical computations, not demonstrations on quantum processors.

The scope of each result must be kept at the scale actually modelled. Sliozberg et al. [6] calculated DGEBA binding to Ti₂CTₓ and Ti₃C₂Tₓ surfaces with DFT; deformation and fracture were investigated separately with coarse-grained MD and compared with manufactured composites. Ehlers et al. [47] used molecular reaction models to compare epoxy–amine pathways and activation energies under isolated, promoted and catalysed conditions. Such calculations inform local chemistry; they do not directly predict the curing history or mechanical properties of a complete cross-linked network.

Conventional DFT can be computationally demanding for large polymeric models. Its accuracy also depends on the exchange–correlation approximation, with important limitations for some strongly correlated, excited-state and dispersion-dominated problems [44]. The size and configurational complexity of a cross-linked network create additional modelling and sampling costs; these are distinct from a functional error. Multiscale approaches offer ways to connect local electronic calculations with larger-scale models [39,40,41].

Quantum solvers could complement this classical baseline for selected electronically correlated active spaces, but they do not automatically improve a DFT prediction. VQE has been demonstrated for small molecular electronic-structure problems [18], while chemically demanding QPE calculations require substantially greater fault-tolerant resources [12,22]. A polymer application must establish that the chosen basis, active space, environment and measured observable address a specific limitation of the classical reference. Table 2 separates demonstrated classical DFT results from the larger-scale properties to which they may contribute.

### 3.3. Molecular Dynamics

MD is considered here as the atomistic bridge between local interactions and polymer-scale behaviour. Predictions depend on the force field, resolution, sampling and boundary conditions [48,49], while parallel algorithms enable efficient treatment of short-range interactions [50]. Reactive potentials can permit bond rearrangements: the original ReaxFF study demonstrated this approach for hydrocarbons [51]. Transfer to a specific polymer chemistry or curing process requires additional validation. For example, the CNT–epoxy study in Table 2 combined DFT and MD [46], while the MXene–epoxy study used coarse-grained MD for deformation and fracture [6]. These examples do not imply that DFT alone resolves nanofiller dispersion or macroscopic failure.

Accessible MD length and time scales depend on model resolution, interaction potentials, algorithms and computing resources [48,49,50]. Direct simulation of engineering-scale structures or long-term degradation generally requires further coarse-graining or coupling across scales [39,40,41]. Force-field transferability is a separate limitation: a potential suitable for one composition or thermodynamic state may not accurately represent another chemical or interfacial environment.

Classical multiscale methods couple atomistic and continuum descriptions through appropriate information transfer and homogenization [39,40,41]. Materials informatics can provide complementary surrogate models and data-driven selection strategies [8,9,10,11]. Quantum calculations are a prospective source of selected local electronic information [12,18,22]; whether they improve a polymer MD model must be established through accuracy, transferability and end-to-end cost comparisons.

### 3.4. Finite Element Analysis

FEA is retained as the continuum-scale component of the multiscale workflow, with emphasis on how lower-scale information and optimization outputs can inform composite-level analysis rather than on standard finite-element formulation [52,53,54].

FEA solves a specified continuum boundary-value problem using chosen constitutive relationships and numerical discretizations [52,53,54]. For composites, predictions of stiffness, damage or interfacial failure therefore depend on appropriate material models, parameters and validation. Multiscale mechanics provides strategies for connecting fine-scale responses to continuum descriptions [39,40,41]. Transfer to a specific composite requires consistent scale coupling and validation of the resulting constitutive description.

Despite its widespread application, FEA possesses several inherent limitations. The predictive capability of continuum-scale simulations depends strongly on the accuracy of constitutive models, material properties, and boundary conditions. Material parameters are often obtained from experimental measurements or lower-scale computational methods, making the reliability of FEA closely dependent on the quality of these input data. Furthermore, conventional FEA cannot directly describe electronic interactions, molecular conformations, or chemical reactions, thereby necessitating integration with atomistic and quantum-mechanical modelling techniques [39,40,41,52,53,54].

Emerging quantum computing technologies may complement finite element modelling indirectly rather than replacing conventional numerical solvers. Gate-based QAOA and Ising/QUBO formulations have been investigated for combinatorial optimization, and related formulations may be relevant to discrete structural design, inverse material identification, and topology-optimization subproblems [29,30,31]. However, these applications remain at an early stage and require comparison with strong classical optimizers before any computational advantage can be claimed.

**Figure 4:**
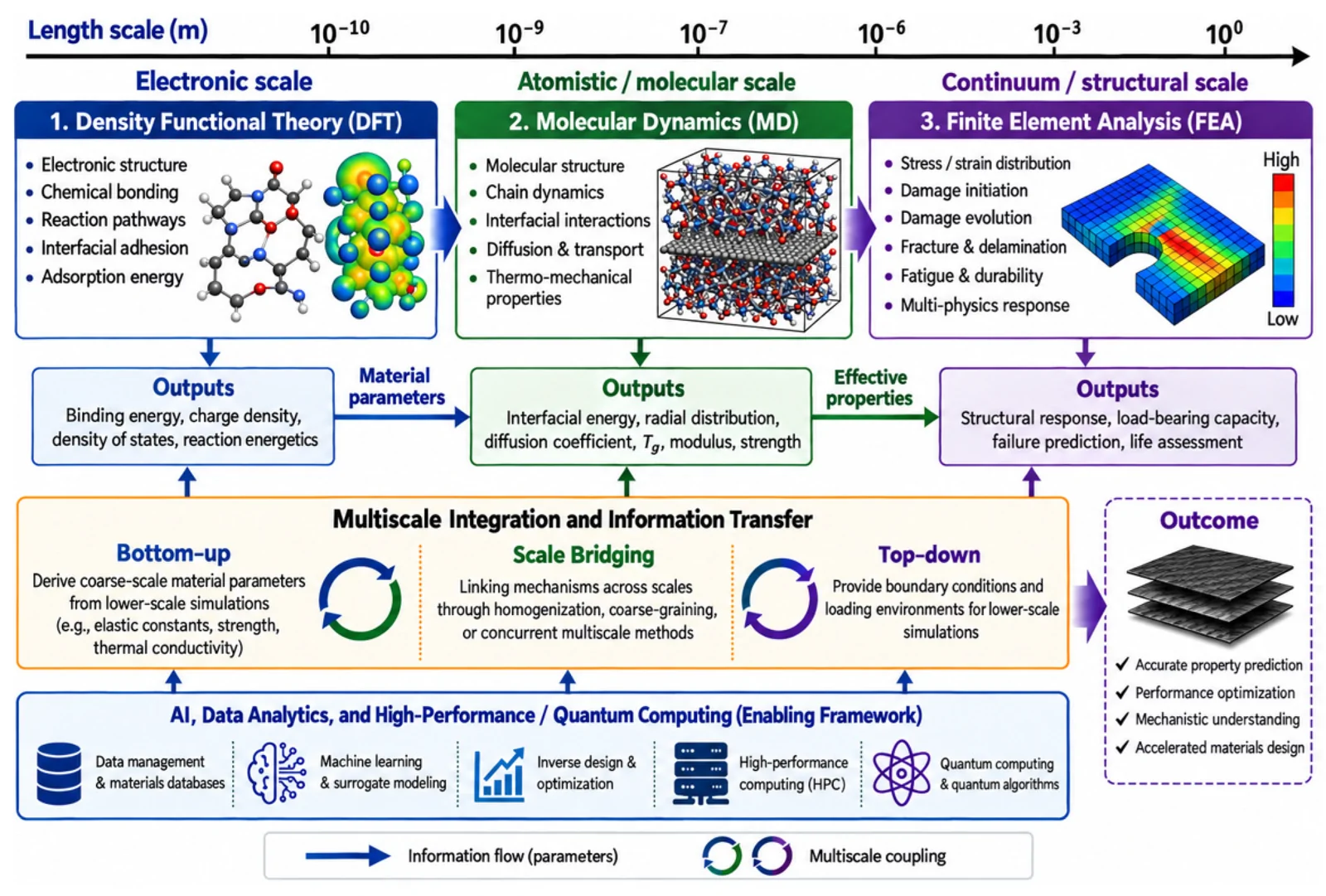
Multiscale integration of density functional theory, molecular dynamics, and finite element analysis for polymeric composite modelling.

Overall, FEA remains the cornerstone of continuum-scale computational modelling for polymeric composites. Future advances may arise from tighter integration with atomistic simulations, artificial intelligence, and quantum computing, enabling more accurate multiscale predictions and accelerating the development of high-performance, multifunctional, and sustainable composite materials. Figure 4 summarizes the hierarchical relationship among the principal computational methodologies employed in polymeric composite modelling.

### 3.5. Artificial Intelligence and Materials Informatics

AI and materials informatics are discussed primarily as data-driven components of hybrid workflows for property prediction, inverse design, and uncertainty reduction rather than as standalone computational methods [8,9,10,11].

Materials informatics provides data-driven property prediction, high-throughput screening and inverse-design strategies across molecular and materials science [8,9,10,11]. In a polymer-composite workflow, composition, reinforcement geometry and processing variables can serve as candidate inputs, with mechanical or functional properties as targets. Applying these methods requires representative training data and independent validation for the intended polymer system and observable.

It should be noted that AI-based methodologies still remain dependent on the availability of high-quality datasets. Experimental uncertainties, inconsistent data formats, limited sample sizes, and incomplete reporting continue to restrict model reliability and generalization. Moreover, many machine learning models operate as “black-box” predictors, providing limited physical interpretability regarding the underlying mechanisms governing material behaviour. These limitations motivate interpretable descriptors, uncertainty assessment and hybrid approaches that combine data-driven learning with physical modelling [8,9,10,11].

Multiscale mechanics supplies methods for transferring information between electronic, atomistic and continuum descriptions [39,40,41]. Materials informatics offers complementary data-driven modelling and optimization tools [8,9,10,11]. Combining these components, and ultimately a quantum solver, into an integrated polymer-composite workflow is a research direction whose accuracy, interoperability and total computational cost must be evaluated.

QML explores possible quantum benefits for selected learning tasks [19]. Such benefits depend on data access, model structure, training and measurement costs; they cannot be inferred from Hilbert-space dimension alone. Polymer-property studies discussed in Section 4.4 provide specific examples, while integration into autonomous polymer-composite discovery platforms remains prospective. Improved predictive accuracy, computational efficiency and experimental outcomes must be demonstrated independently.

Overall, AI should be regarded not as a replacement for conventional computational modelling but as an enabling technology that complements DFT, MD, and FEA by improving prediction accuracy, accelerating optimization, and facilitating data-driven materials discovery. The continued integration of AI with quantum computing and computational materials science may play a central role in the future development of intelligent polymeric composite design and digital materials engineering. Table 3 summarizes representative AI techniques together with their candidate applications, representative outputs, and methodological capabilities.

## 4. Applications of Quantum Computing in Polymeric Composites

To distinguish evidence levels, the applications in this section are classified as: (i) polymer-specific demonstrations, with quantum-hardware execution, classical simulation of quantum circuits, and numerical validation of composite designs identified separately; (ii) demonstrations on generic molecular or materials systems whose transfer to polymers remains to be validated; (iii) simplified algorithmic benchmarks; or (iv) prospective applications. A simplified lattice-polymer model can establish polymer-specific feasibility without representing an atomistic engineering composite. These classifications describe the scope and mode of evidence, not a ranking of quantum advantage.

Concrete polymer-specific studies anchor the discussion. Micheletti et al. investigated lattice-polymer sampling [35]; Cho and Cheon addressed Cu-film placement in CFRP [36], followed by natural-fibre laminate optimization by Cho et al. [37]; Ishiyama et al. tested polymer-property learning on IonQ hardware [55]; and Zhang et al. evaluated the PolyQT architecture computationally [56]. Table 4 provides a concise comparison of their evidence levels. The distinction between a working quantum-compatible workflow, physical materials validation, and an end-to-end computational advantage is maintained throughout this review.

Evidence levels describe the reported execution and validation, not proof of quantum advantage. General computational advantage requires matched classical baselines and end-to-end cost comparisons. Generic-material electronic-structure benchmarks [32,33,34] are discussed separately and do not establish polymer-specific validation.

### 4.1. Quantum Computing for Electronic Structure Calculations

The scaling of classical electronic-structure methods depends strongly on the level of theory. Hartree–Fock and most standard Kohn–Sham DFT implementations have polynomial formal scaling, whereas exact full configuration interaction grows combinatorially/exponentially with the number of electrons and orbitals; systematically improvable correlated wavefunction methods can also carry steep polynomial prefactors. Quantum algorithms should therefore not be interpreted as competing with an “exponentially scaling DFT” baseline. Their potential relevance is instead most credible for selected correlated-electron subproblems that become difficult for conventional approximations [12,18,22,42,43,44].

Electronic-structure calculations constitute the foundation for understanding the physicochemical behaviour of polymeric composites because interfacial adhesion, chemical bonding, charge transfer, adsorption, and reaction mechanisms originate from quantum-mechanical interactions. Conventional DFT has become the standard computational approach for investigating these phenomena; however, its computational cost increases rapidly with molecular complexity, particularly for highly cross-linked polymer networks, multifunctional nanocomposites, and large interfacial systems. Quantum computing is expected to complement rather than replace DFT by enabling more accurate electronic-structure calculations for complex molecular systems that remain computationally challenging using classical approaches [18,22,42,43,44].

VQE has demonstrated accurate ground-state energies for small molecular benchmarks, but its practical cost includes large numbers of circuit measurements, ansatz-dependent circuit depth, sensitivity to hardware noise, and non-convex classical optimization. These overheads can dominate end-to-end runtime, and a general quantum advantage for chemically relevant systems has not been demonstrated [18,20,21,33]. Accordingly, VQE results for small molecules should be treated as algorithmic demonstrations rather than direct evidence of scalable polymer-composite simulation.

QPE can in principle provide systematically improvable eigenvalue estimates on fault-tolerant hardware, but “near-exact” accuracy applies only within a specified basis and active space. Application to realistic polymer systems would additionally require feasible state preparation, logical-qubit counts, fault-tolerant gate counts, error correction, and an active-space or embedding strategy. Thus, QPE for polymer applications is better viewed as a longer-term prospective solver for localized electronically active regions than as a route to direct treatment of entire large macromolecules [12,22,32,38].

Potential polymer targets for quantum electronic-structure calculations include selected interfacial bonds, adsorption sites and curing reaction centres. The classical studies in Table 2 establish the relevance of these local questions [6,45,46,47], but do not demonstrate that a quantum processor can predict adhesion or composite performance at engineering scales. Transfer requires a justified active space and validation for each chemical environment.

Recent materials benchmarks clarify both what has been demonstrated and what remains prospective for polymeric systems. The CP2K–Qiskit Nature embedding implementation of Battaglia et al. [32] used VQE-based active-space calculations for a periodic materials defect, whereas BenchQC [33] quantified how ansatz choice, optimizer, basis set, simulator, and noise assumptions affect VQE energy predictions. Barroca et al. [34] further demonstrated sample-based quantum diagonalization for a surface reaction on present-day IBM hardware. These studies provide transferable algorithms and software patterns for polymer-interface electronic-structure problems, but they should not be presented as direct demonstrations on realistic polymer chains or fibre–matrix interfaces. Published quantum electronic-structure calculations at engineering-relevant polymer length scales remain scarce; therefore, the polymer applications described here are best interpreted as scientifically motivated targets for methods already benchmarked on smaller molecular and materials systems.

The origin of any future performance advantage must also be distinguished from simple hardware scaling. Increasing the number of physical qubits enlarges the representable problem only if fidelity, connectivity, coherence, and error correction are sufficient; fault-tolerant calculations ultimately depend on logical qubits, which can require substantial physical-qubit overhead. At the same time, algorithmic innovations—including active-space selection, Hamiltonian compression, symmetry reduction, ansatz design, circuit-depth reduction, error mitigation, efficient measurement, and quantum–classical decomposition—can reduce the quantum resources required for a fixed scientific problem [12,20,22,32,33,34,38]. VQE, for example, trades circuit depth for repeated measurements and classical optimization, whereas QPE offers stronger asymptotic precision but requires fault-tolerant resources. Consequently, neither a larger physical-qubit count nor a more sophisticated algorithm alone guarantees practical quantum advantage; credible comparisons require matched classical baselines, solution accuracy, scaling behaviour, and total end-to-end computational cost.

However, current quantum processors are constrained by limited qubit numbers, gate errors, decoherence, and restricted circuit depth, which limit the size and complexity of molecular systems that can be accurately simulated. Consequently, hybrid quantum–classical workflows integrating quantum algorithms with DFT, molecular dynamics, artificial intelligence, and high-performance computing are expected to dominate near-term applications while quantum hardware continues to mature [12,18,19,22].

**Figure 5:**
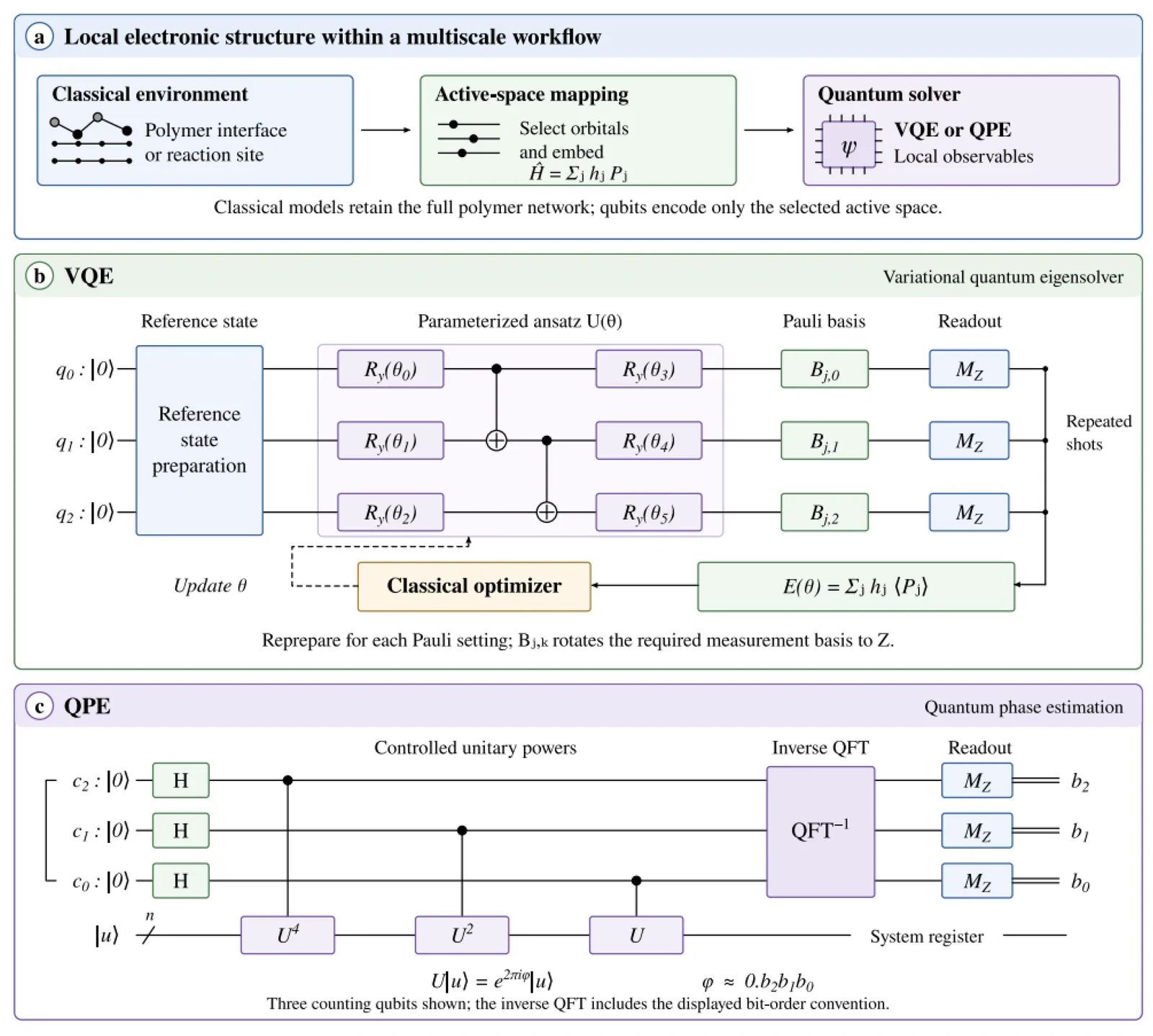
Localized quantum electronic-structure workflow and illustrative circuits. (**a**) Classical embedding and active-space mapping. (**b**) VQE with reference preparation, a parameterized ansatz, Pauli-basis changes, repeated measurements and a classical energy-minimization loop. (**c**) QPE with controlled unitary powers, inverse QFT and counting-register measurements.

Figure 5 separates the multiscale workflow from two distinct electronic-structure algorithms. In panel (a), classical models define a localized reaction centre or interfacial active space and retain the surrounding polymer and reinforcement environment. After fermion-to-qubit mapping, the quantum solver returns selected observables for validation and transfer to the classical model. The circuit panels illustrate algorithmic operations; they do not encode a complete composite or reproduce a particular published implementation [18,22,25,32].

In Figure 5b, reference-state preparation is followed by an illustrative parameterized ansatz, measurement-basis rotations and computational-basis readout. Each Pauli-string expectation value requires repeated state preparation and sampling; the weighted expectations form the VQE energy supplied to the classical optimizer. The basis change is the identity for Z, a Hadamard gate for X, or a phase-dagger gate followed by a Hadamard gate for Y. Measuring every qubit only in the computational basis is insufficient for a general electronic Hamiltonian. The three-wire ansatz is pedagogical and is not asserted to conserve particle number or spin; a chemical calculation must choose an appropriate ansatz and enforce the required symmetries [18,20,22].

Figure 5c shows QPE with three illustrative counting qubits initialized in zero states and placed in superposition by Hadamard gates. Controlled powers of the same unitary act on a separate system register, followed by an inverse quantum Fourier transform and measurement of the counting register. For an eigenstate input, the measured phase is converted to energy using the specified Hamiltonian evolution time, energy shift and phase-unwrapping range. For an approximate input, eigenphases are sampled according to the input overlaps. Three counting qubits illustrate the topology only and do not imply chemically useful precision or a polymer resource estimate [22,25].

Quantum-derived local energies or response quantities must be transferred back with consistent boundary conditions and uncertainty estimates [32,57]. Charge distributions require additional observables, reaction barriers require calculations at multiple geometries, and finite-temperature free-energy barriers require further statistical-mechanical treatment. Adhesion, cross-linking and bulk mechanical properties are therefore not direct outputs of a single energy measurement.

### 4.2. Quantum-Assisted Molecular Simulations

Quantum-assisted molecular simulations represent an emerging research direction that integrates quantum computing with classical atomistic modelling to overcome the computational limitations associated with conventional MD simulations. While classical MD has become an indispensable tool for investigating polymer chain dynamics, interfacial interactions, diffusion behaviour, and thermomechanical properties of polymeric composites, its predictive capability is fundamentally constrained by the accuracy of empirical force fields and the computational cost associated with large molecular systems [48,49,50]. Hybrid quantum–classical approaches offer a promising strategy to improve atomistic simulations by incorporating quantum-mechanical information into molecular modelling workflows [12,22,57].

Conventional MD propagates atomic motion using interatomic potentials, with predictions depending on model choice and parameterization [48,49,50]. ReaxFF provides a reactive formulation originally demonstrated for hydrocarbons [51]; its use for a polymer–nanofiller interface requires validation for that chemical environment. Machine-learned potentials can be fitted to electronic-structure data, with reliability determined by training coverage and validation [58,59].

A small number of VQE electronic energies is not sufficient to define a transferable polymer force field or potential-energy surface. A practical workflow would require many representative configurations spanning conformational, interfacial, and—when relevant—reactive space; consistent energies and preferably forces/gradients; uncertainty control; and validation against configurations excluded from fitting. Quantum calculations could therefore supply selected high-value training points within an active-space or embedded region, while classical electronic-structure calculations and machine-learned potentials provide the dense sampling needed for MD [57,58,59,60,61,62].

For polymeric composites, this requirement is especially stringent because cross-linking, weak dispersion, charge redistribution, and heterogeneous interfaces generate many chemically distinct local environments. Quantum-derived potentials should therefore be described as a prospective multilevel data source rather than as a direct consequence of obtaining a few quantum energies.

The classical interface and curing studies in Table 2 provide concrete starting points for prospective quantum-assisted molecular simulation [6,45,46,47]. They identify local binding and reaction questions for which a validated quantum solver might eventually supply additional information. Extending these results to curing kinetics, cross-link density, residual stress or durability would still require reaction sampling, kinetic modelling and appropriate MD or continuum calculations. Established machine-learned potentials, including DeePMD-related models, ANI and GAP, demonstrate how classical electronic-structure data can support efficient atomistic simulations [58,59,60,61,62]. Their performance is not evidence of quantum-processor execution, and a transferable polymer potential trained on quantum-processor outputs remains to be demonstrated.

A concrete polymer-specific quantum-computing example was reported by Micheletti et al. [35], who represented lattice-polymer ensembles with interacting binary tensors and solved the resulting discrete energy-minimization problems using a D-Wave quantum annealer. Their formulation accommodated self-avoidance, branching, and looping and was used to sample polymer mixtures from low to high density. Importantly, this work addressed statistical-mechanical sampling rather than ab initio electronic structure. It nevertheless demonstrates how a polymer problem can be reformulated for quantum hardware and highlights that practical capability arises from the combination of problem encoding, algorithm, and hardware architecture rather than from qubit number alone.

However, several technical challenges remain before quantum-assisted molecular simulations become practical for engineering-scale polymeric composite research. Current quantum hardware continues to suffer from limited qubit numbers, gate errors, decoherence, and restricted circuit depth, while hybrid quantum–classical algorithms require efficient coupling between quantum processors and conventional high-performance computing infrastructures [12,22,57]. The studies reviewed here do not demonstrate a transferable force field trained on quantum-processor outputs and validated for highly cross-linked, multifunctional polymer systems. Overall, quantum-assisted molecular simulations should be regarded as a complementary extension of conventional molecular dynamics rather than a replacement. By combining quantum-mechanical accuracy with classical atomistic efficiency, hybrid computational frameworks have the potential to improve molecular-level understanding of polymeric composites and accelerate the development of next-generation multifunctional materials.

### 4.3. Quantum Optimization for Composite Design

Quantum optimization has emerged as a promising route for selected engineering-design and materials-search problems, including constituent selection, fibre architecture, nanofiller concentration, curing schedules, and manufacturing parameters. However, quantum superposition should not be interpreted as a brute-force evaluation of all candidate designs at once. Potential benefit instead depends on how the engineering objective and constraints are encoded into an Ising or QUBO Hamiltonian, how effectively the quantum evolution or variational circuit explores that landscape, and how quantum processing is integrated with classical preprocessing and postprocessing [29,30,31]. Thus, algorithmic formulation, embedding overhead, noise, sampling requirements, and solution quality are as important as raw qubit count when assessing quantum optimization for polymeric composites.

Among the available quantum optimization techniques, the QAOA has attracted considerable attention owing to its compatibility with NISQ hardware. QAOA combines parameterized quantum circuits with classical optimization routines to solve combinatorial optimization problems and has been investigated on combinatorial benchmarks such as MaxCut [29,30]. In this review, gate-based QAOA for realistic polymer-composite design remains prospective. The demonstrated annealing studies [36,37] do not establish QAOA performance, and suitability for multi-objective composite design must be assessed through explicit encodings and matched classical benchmarks.

Another promising direction involves quantum annealing, which provides a framework for discrete optimization through quantum dynamics. Quantum annealers search for candidate solutions to problems mapped onto Ising or Quadratic Unconstrained Binary Optimization (QUBO) formulations; this mapping alone does not establish computational efficiency relative to classical solvers [31,63]. In polymeric composite engineering, such formulations may facilitate optimization of laminate stacking sequences, fibre orientations, additive manufacturing toolpaths, and topology optimization, thereby supporting the development of lightweight and multifunctional composite structures.

The optimization paradigms should also be distinguished. QAOA is a gate-based variational algorithm; physical quantum annealing uses analog annealing hardware; simulated annealing is a classical stochastic optimizer; and “quantum-inspired” methods are classical algorithms motivated by quantum formulations. For polymer/composite QUBO problems, assessment should report logical-variable count, embedding overhead and connectivity, constraint penalties, solution quality, sampling budget, and wall-clock cost, and should compare against appropriate classical baselines such as simulated annealing, mixed-integer optimization, evolutionary algorithms, or other problem-specific solvers [29,30,31,36,37,63].

A direct polymeric-composite application was demonstrated by Cho and Cheon [36] for a CFRP hybrid composite containing thin Cu films. Correlations between laminate plies and Cu-film placement were first obtained from finite element analysis, after which the placement problem was formulated as a QUBO model and implemented with the Ocean SDK and D-Wave Leap. Quantum annealing was then used to identify Cu-film arrangements that maximize through-thickness thermal conductivity. This study provides a concrete example of a hybrid FEA–quantum workflow in which the quantum component addresses a discrete design subproblem rather than replacing the underlying physics-based simulation.

The approach was extended in 2025 by Cho et al. [37] to natural-fibre hybrid composites. Their stacking-sequence problem comprised 114 design variables generated from six natural fibres and 19 orientation angles; the objective was converted to a QUBO formulation, optimized by quantum annealing under specific-gravity and cost constraints, and the resulting laminates were subsequently assessed by Charpy impact simulation. The Ramie/Jute configuration produced the highest simulated impact-energy absorption among the reported candidates. The reported impact result is a numerical assessment, not an experimentally measured improvement in manufactured laminates. These case studies demonstrate application-specific quantum-compatible formulations for polymeric composites, but they do not by themselves establish universal quantum speedup. Such a claim requires controlled comparisons with state-of-the-art classical optimizers using identical constraints, accuracy targets, scaling regimes, and wall-clock accounting.

Beyond the demonstrated QUBO/annealing case studies in CFRP and natural-fibre laminates [36,37], broader inverse design of polymeric composites remains prospective. Any claimed benefit from QAOA or annealing should therefore be reported relative to matched classical comparators and identical constraints, accuracy targets, and end-to-end timing, rather than inferred from superposition or the use of quantum hardware alone.

### 4.4. Quantum Machine Learning for Polymeric Composites

QML represents an emerging research area that combines quantum computing with artificial intelligence to enhance data-driven materials discovery and optimization. Unlike conventional machine learning algorithms executed entirely on classical computers, QML utilizes quantum circuits to process information in high-dimensional Hilbert spaces, offering the potential to improve computational efficiency for selected learning and optimization tasks [64,65,66]. Although practical applications remain limited by current quantum hardware, QML has attracted increasing attention as a promising computational paradigm for next-generation materials informatics.

Quantum feature maps, variational classifiers and quantum kernels provide model families for learning nonlinear relationships [64,65,66]. These methods and their selected classification demonstrations motivate applications in materials informatics. Their extension to polymer-composite screening, corrosion resistance, durability or inverse design remains prospective. The polymer-property studies described below provide narrower application evidence; broader claims require independent validation and matched classical baselines.

Polymer-specific QML has progressed beyond generic proposals. Ishiyama et al. [55] trained a four-qubit MERA-type variational model for polymer-property prediction on an actual IonQ ion-trap processor (11 available qubits), using shot-based expectation values. The target glass-transition temperatures were generated by Synthia, and the hardware test covered a limited optimization segment initialized from simulator-trained parameters. The result demonstrates hardware trainability, not independent experimental property validation or a computational speedup. Zhang et al. [56] subsequently introduced the PolyQT hybrid quantum–Transformer model for six polymer properties. Their best configuration used eight qubits and improved several prediction metrics, including under sparse-data conditions, but prediction performance does not establish a physical-hardware speedup. These examples should therefore be classified as demonstrated polymer-data applications, while evidence for hardware-level quantum advantage remains absent.

Recent dequantization results also temper broad claims of QML advantage. Tensor-network and random-feature analyses identify conditions under which variational QML models admit effective classical surrogates [67,68]. Accordingly, polymer-QML studies should compare against strong classical kernels and tensor-network baselines, include data-loading and sampling costs, and reserve the term quantum advantage for cases in which an efficient classical surrogate has been excluded.

Several challenges remain before QML can be routinely applied in polymer science. Current quantum processors are constrained by limited qubit numbers, noise, and decoherence, while the availability of large, standardized datasets suitable for quantum learning remains limited. Consequently, near-term developments are expected to focus on hybrid quantum–classical architectures that combine quantum feature mapping with established machine learning algorithms, enabling gradual integration of QML into computational materials engineering.

**Figure 6:**
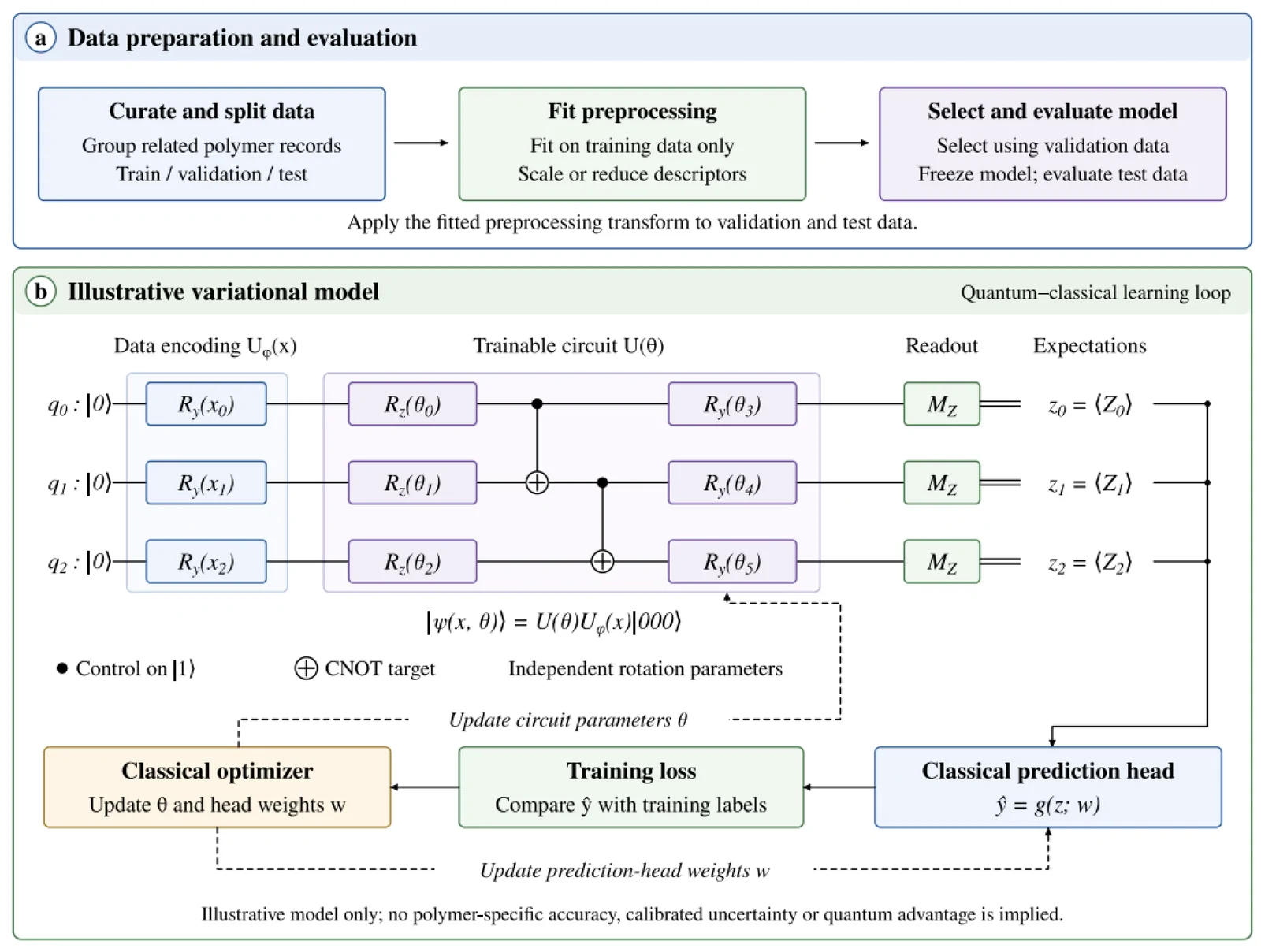
Illustrative variational QML workflow for polymer-property prediction. (**a**) Grouped data splitting precedes training-only preprocessing and model selection. (**b**) Descriptor-dependent encoding *Uφ*(*x*), trainable rotations *U*(*θ*), directed CNOT connections, Z-basis readout and classical prediction and optimization are shown separately.

Figure 6 distinguishes data preparation, the quantum model and classical training. The illustrative feature map uses fixed rotations determined by scaled descriptors x, whereas the subsequent variational layer uses independently trainable angles θ. Its filled controls and circled targets specify two CNOT operations unambiguously. Repeated computational-basis measurements estimate the indicated Z observables; a classical prediction head converts these estimates to a property prediction, and the training loss updates θ and the head weights. This is a variational-model example, not a quantum-kernel circuit or a reproduction of the architectures in the cited polymer studies [55,56].

Data are split into training, validation and test groups before fitting normalization, dimensionality reduction or other learned preprocessing. Related records should be grouped where needed to prevent information leakage. Validation data guide model selection, and the held-out test data are used for final evaluation after the model is fixed. Predictive accuracy, uncertainty calibration, interpretability and computational advantage require separate tests; none follows automatically from a quantum feature map or an expectation-value readout. Quantum-kernel methods instead estimate state overlaps for a classical kernel learner and should be evaluated as a separate model family [65,66]. Table 5 summarizes the comparison of representative demonstrated quantum-computing applications relevant to polymers and materials.

### 4.5. Digital Twins and Autonomous Materials Discovery

Quantum-enabled digital twins and autonomous discovery of polymeric composites are prospective applications. The studies reviewed here do not demonstrate an integrated polymer-composite platform in which a quantum processor improves a closed experimental loop. A digital twin is a virtual representation of a physical material or manufacturing process that continuously integrates computational models with experimental observations and sensor data to predict material behaviour throughout its life cycle [69,70].

Digital-twin frameworks connect computational representations with data from the corresponding physical system [69,70]. A proposed polymer-composite implementation could couple multiscale models, sensing and manufacturing information, but improvements in prediction or control require validation. A quantum solver might eventually address a selected electronic-structure or optimization subproblem [12,22]; any benefit must be measured against a matched classical workflow.

Autonomous materials discovery represents another promising direction in which quantum computing may play a significant role. Autonomous research platforms combine robotics, high-throughput experimentation, artificial intelligence, and computational modelling to perform closed-loop materials optimization with minimal human intervention [71,72]. Within these platforms, machine learning algorithms identify promising candidate materials, computational simulations evaluate their predicted performance, automated synthesis and characterization generate new experimental data, and optimization algorithms iteratively refine material compositions. Adding a quantum solver to such a workflow is a research proposal: its contribution would need to be isolated from the benefits of classical AI, robotics, and automation and assessed using experimental success rates and total time to a validated material.

A prospective integration would connect a selected quantum subproblem with a digital twin, materials database, and experimental feedback loop. Before claims of real-time optimization or adaptive process control are justified, the combined workflow must meet latency, uncertainty, and reproducibility requirements under realistic manufacturing constraints. The classical digital-twin and autonomous-laboratory literature [69,70,71,72] motivates this architecture but does not demonstrate a quantum-enabled polymer-composite implementation.

Several challenges remain before fully autonomous quantum-enabled materials platforms become practical. Current quantum hardware is limited by qubit scalability, noise, and error correction requirements, while digital twin technologies require standardized data formats, interoperable computational workflows, and reliable integration between experimental and computational environments. Furthermore, robust data governance, model interpretability, cybersecurity, and validation against experimental observations will be essential to ensure the reliability of future autonomous materials systems [69,70,71,72].

Overall, the integration of quantum computing, digital twins, and autonomous materials discovery represents a long-term vision for intelligent polymeric composite engineering. Its feasibility and value remain open questions. Progress should be assessed through reproducible demonstrations that connect quantum outputs to experimentally validated composite decisions and compare the complete workflow with a classical alternative.

## 5. Challenges and Future Perspectives

Although quantum computing has demonstrated remarkable progress in quantum chemistry, optimization, and machine learning, its practical application to polymeric composite research remains in its infancy [12,18,22]. Recent assessments have emphasized that the successful deployment of quantum technologies will require continued advances in scalable quantum hardware, efficient quantum algorithms, software infrastructure, and interdisciplinary collaboration among quantum scientists, computational materials researchers, and polymer engineers [13,73]. Consequently, quantum computing should currently be regarded as a complementary computational technology rather than a replacement for established modelling approaches.

Recent studies support a problem-specific view of progress: active-space embedding and electronic-structure benchmarks [32,33,34] address local quantum chemistry, composite-design studies [36,37] address discrete engineering decisions, and polymer-learning models [55,56] address selected datasets. These results concern different backends, validation levels and cost measures, so they cannot be combined into a single claim of composite-scale quantum advantage. Table 4 and Table 5 retain those distinctions; broader materials-design proposals [24] identify research directions rather than additional polymer demonstrations.

One of the most significant challenges is the limited capability of current NISQ hardware. Existing quantum processors are constrained by limited qubit numbers, finite coherence times, gate errors, and restricted circuit depths, which significantly limit the size and complexity of polymeric systems that can be accurately simulated [13]. Although hybrid quantum–classical algorithms, including the VQE and the QAOA, have partially mitigated these limitations [18,29,30], realistic simulations of highly cross-linked polymer networks, multifunctional nanocomposites, and engineering-scale composite structures remain beyond the capabilities of current quantum devices [12,22]. Recent studies further suggest that quantum-centric supercomputing, where quantum processors operate as accelerators within classical HPC systems, represents one of the most promising pathways toward practical quantum-enabled computational materials science [23].

An order-of-magnitude resource estimate helps calibrate these polymer applications. In common second-quantized encodings, the qubit count scales roughly with the number of spin orbitals before symmetry reduction, so an early fault-tolerant regime of approximately 25–100 logical qubits corresponds to localized active spaces containing only tens of spatial orbitals rather than an entire epoxy network or graphene–polymer interface [74]. For one compiled minimal H2 QPE example, Blunt et al. estimated approximately 1000 physical qubits and 2300 quantum-error-correction rounds [75]. This is a circuit-specific estimate, not a general resource requirement for chemical accuracy. Recent surface-code experiments have demonstrated operation below the error-correction threshold [76], but a physical gate fidelity is not itself a chemistry-level logical error budget. Long algorithms still require sufficiently low effective logical error rates and an explicit assessment of error-correction overhead. Thus, the realistic target for polymer composites is a localized reaction or interfacial active region embedded in a classical environment, not direct quantum treatment of the full composite.

Another important challenge arises from the inherently multiscale nature of polymeric composites. Their macroscopic performance is governed by phenomena occurring across quantum, atomistic, mesoscale, and continuum length scales [39,40,41,42,43,44,48,49,50,52,53,54]. Consequently, future computational frameworks should emphasize seamless integration of DFT, MD, FEA, AI, and quantum computing to establish robust multiscale modelling platforms. Efficient information transfer between these computational levels will be essential for accurately predicting structure–property relationships while maintaining computational efficiency [39,40,41].

Polymeric composites also introduce barriers that are more specific than generic NISQ hardware limitations. Repeat-unit growth and conformational disorder rapidly enlarge electronic active spaces; cross-linked thermosets and heterogeneous fibre–matrix or nanofiller interfaces require simultaneous treatment of covalent bonding, weak interactions, and many local environments; and discrete composite-design problems can require large QUBO embeddings as the number of plies, orientations, constituents, and manufacturing constraints increases. In addition, quantum-derived local information must be transferred consistently to force fields, mesoscale models, and continuum constitutive laws. These issues make polymer validation, classical baseline selection, and error propagation across scales central technical requirements rather than secondary implementation details [35,36,37,39,40,41,44,48,49,50].

Reliable, representative and well-documented datasets are important for materials learning, digital twins and autonomous experimentation [55,69,70,71,72]. Dataset size alone does not ensure reliable predictions, and some polymer-learning studies explicitly address sparse-data conditions [56]. Inconsistent descriptors, test conditions or provenance can hinder comparison and generalization. FAIR (Findable, Accessible, Interoperable, and Reusable) data principles provide a framework for data stewardship [77]; adopting these principles alongside standardized reporting is recommended for future quantum-enabled materials informatics.

Experimental validation should target the observable predicted by the model. Depending on the application, appropriate comparisons may involve spectroscopy, interfacial measurements, thermal characterization or mechanical testing. Feedback between models and measurements is central to classical digital twins and autonomous experimentation [70,71]. Extending this strategy to quantum-assisted polymer modelling will require application-specific validation of both local quantum predictions and their transfer to composite properties.

**Figure 7:**
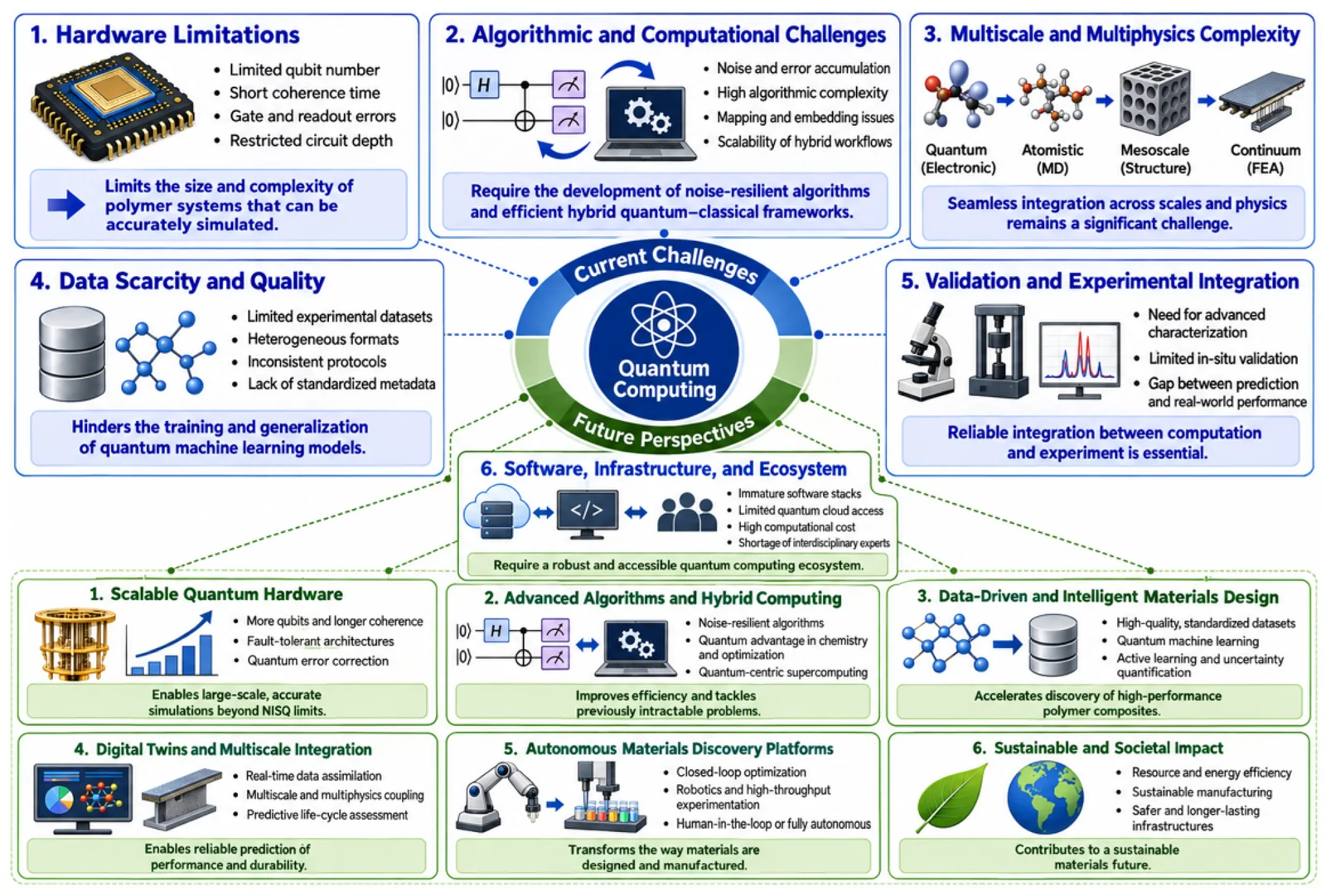
Current challenges and future perspectives for a robust and accessible quantum computing ecosystem.

Future research should test whether a quantum solver can improve a clearly defined polymer-composite subproblem within an otherwise classical workflow. Priority targets include localized electronic active spaces, constrained laminate design, and polymer-property learning, with the execution backend and evidence level reported explicitly. Quantum-enabled digital twins and autonomous laboratories remain longer-term proposals, motivated by classical developments [69,70,71,72]. Their practical value will depend on reproducible gains in accuracy, solution quality, or end-to-end cost and on successful experimental validation, rather than on hardware progress alone [12,13,22,23,73]. Figure 7 summarizes the technical challenges and prospective research directions; it is not evidence of an implemented integrated platform.

## 6. Conclusions

This review assessed quantum computing as a possible complement to established methods for polymeric composites. The available evidence is uneven: polymer-specific studies demonstrate selected lattice-sampling, discrete-design, and property-learning tasks, while many electronic-structure and multiscale applications are supported only by transferable non-polymer benchmarks or remain prospective. Algorithmic feasibility, execution on quantum hardware, and practical quantum advantage are therefore distinct conclusions.

The clearest polymer-specific evidence includes lattice-polymer sampling, QUBO-based composite design with numerical evaluation, and limited hardware training of a polymer-property model. Computational polymer-learning studies provide additional evidence of model performance under their stated conditions. As summarized in Table 4, these results support task-specific proof of concept, not direct quantum simulation of a complete engineering composite or a general end-to-end advantage over strong classical methods. Numerically predicted composite properties must also be distinguished from measurements on manufactured materials.

For electronic-structure and molecular-simulation applications, a testable research direction is to embed a localized quantum calculation within DFT, MD, or other classical models. Quantum-processor-derived polymer force fields, realistic interfacial reaction predictions, and quantum-enabled autonomous discovery remain prospective within the literature reviewed here. Their assessment requires appropriate active spaces, representative configurations, uncertainty propagation, and validation against independent classical and experimental references. 

Further work should prioritize reproducible polymer-specific benchmarks that report accuracy, scaling, data-loading and measurement costs, classical optimization overhead, and total execution time. Hybrid workflows may offer useful capabilities for selected problems if these tests establish a measurable benefit. The present evidence does not support a reliable timeline for routine industrial deployment or a general claim of quantum speedup in polymeric composite engineering.

## Figures and Tables

**Table 1:** Comparison of the principal computational methods employed in polymeric composite modelling.

Method	Typical Length Scale	Primary Capability	Representative Applications	Major Limitations	Potential Synergy with Quantum Computing
Density Functional Theory (DFT)	10^−10^–10^−9^ m	Electronic structure, chemical bonding, reaction mechanisms	Interfacial bonding, nanofiller functionalization, adsorption, polymerization	High computational cost for large systems	Quantum algorithms (VQE, QPE) may improve electronic-structure calculations
Molecular Dynamics (MD)	10^−9^–10^−7^ m	Atomistic dynamics, diffusion, interfacial behaviour	Polymer chain conformation, curing, degradation, filler dispersion	Limited time and length scales; force-field dependence	Quantum-derived force fields and hybrid QM/MM simulations
Finite Element Analysis (FEA)	10^−5^–10^1^ m	Continuum-scale structural analysis	Stress analysis, failure prediction, fatigue, durability	Requires constitutive models and material parameters	Improved constitutive models informed by quantum and atomistic simulations
Artificial Intelligence/Machine Learning (AI/ML)	Data-driven	Property prediction, optimization, inverse design	Materials discovery, process optimization, performance prediction	Requires representative, high-quality data; interpretability varies	QML as a candidate model; benefit requires benchmarking
Quantum Computing	Multiple scales (algorithm dependent)	Quantum simulation, optimization, quantum machine learning	Electronic structure, reaction pathways, optimization, molecular modelling	Current hardware noise, limited qubits, immature software ecosystem	Complements classical methods through hybrid quantum–classical workflows

**Table 2:** Primary studies using classical DFT for polymer interfaces and epoxy curing.

Application	System Actually Modelled	Supported Result and Scope	Source
Graphene and graphene oxide adhesion	DGEBA/DDS cured-epoxy model on graphene or graphene oxide	Dispersion-corrected DFT of adhesion and orbital interactions; local interface model.	[45]
CNT interface and functionalization	Single-walled CNTs and DGEBA; varied tube structures and surface groups	DFT and MD assessment of binding and interface behaviour; results depend on the model and method.	[46]
MXene–epoxy binding	DGEBA on Ti_2_CT_x_ and Ti_3_C_2_T_x_ with specified surface terminations	DFT binding and electronic interactions; fracture and deformation assessed separately by coarse-grained MD.	[6]
Epoxy–amine curing chemistry	Molecular epoxy–amine reaction models under isolated, promoted and catalysed conditions	DFT pathways and activation energies; local reaction chemistry, not full-network curing kinetics.	[47]

**Table 3:** Candidate roles of AI methods in polymer-composite workflows, informed by general materials-ML literature [8,9]. These entries describe methodological capabilities rather than comparative performance benchmarks.

AI Technique	Typical Application	Representative Output	Methodological Capability
Artificial Neural Networks (ANN)	Prediction of mechanical and thermal properties	Elastic modulus, tensile strength, thermal conductivity	Captures highly nonlinear structure–property relationships
Random Forest (RF)	Property prediction and feature importance analysis	Composite performance, feature ranking	Ensemble prediction and feature-importance estimates
Gaussian Process Regression (GPR)	Bayesian optimization and surrogate modelling	Processing parameter optimization, uncertainty estimation	Prediction with model-based uncertainty estimates
Support Vector Machine (SVM)	Classification and regression	Failure mode classification, durability prediction	Kernel-based classification or regression
Deep Learning (DL)	Microstructure characterization and image analysis	Damage detection, crack segmentation, defect identification	Automated feature extraction from large datasets
Convolutional Neural Networks (CNN)	Microscopy and microstructure image analysis	Fiber orientation, pore detection, crack recognition	Learning spatial features from images
Graph Neural Networks (GNN)	Molecular and polymer structure learning	Molecular property prediction, polymer representation	Direct modelling of graph-based molecular structures
Reinforcement Learning (RL)	Autonomous process optimization	Manufacturing parameter optimization	Adaptive sequential decision-making
Generative Artificial Intelligence	Inverse materials design	Novel polymer formulations and composite architectures	Generation of candidate materials for evaluation
Quantum Machine Learning (QML)	Molecular property prediction and optimization	Polymer-property prediction on selected datasets	Task-dependent potential; general speedup not demonstrated

**Table 4:** Polymer-specific quantum-computing studies and the scope of their evidence.

Study and Polymer Target	Method and Execution	Evidence Level	What the Evidence Supports and Limits
Micheletti et al. [35] Lattice-polymer mixtures	Discrete encoding; D-Wave annealing	Hardware proof of concept on a simplified polymer model	Polymer sampling feasibility; not atomistic simulation of a realistic composite.
Cho and Cheon [36] Cu-film placement in CFRP	FEA-derived QUBO; D-Wave workflow	Composite-design proof of concept	Conductivity-oriented design with numerical evaluation; general quantum speedup not established.
Cho et al. [37] Natural-fibre hybrid laminates	QUBO annealing; classical laminate and impact models	Composite-design study with numerical validation	114 design variables; simulated impact response, not measured laminate improvement.
Ishiyama et al. [55] Polymer-property prediction	Four-qubit variational model; simulator and IonQ	Limited hardware training demonstration	Learning on computed polymer-property data; not a full hardware training or experimental validation study.
Zhang et al. [56] Polymer-property prediction	PolyQT quantum–Transformer computational model	Computational polymer-data study	Prediction benchmark; no physical quantum-hardware advantage demonstrated.

**Table 5:** Quantitative comparison of representative demonstrated quantum-computing applications relevant to polymers and materials.

Study/Maturity	System/Target	Algorithm/Encoding	Backend/Qubits/Ansatz	Classical Reference/Accuracy	Cost/Quantum Advantage
Battaglia et al. [32] Generic-material demonstration	MgO oxygen-vacancy optical states	Active-space embedding + VQE/qEOM	CP2K–Qiskit workflow; qubits depend on selected active space	Compared with ab initio/experiment; competitive embedded-spectrum results	No general quantum advantage demonstrated; embedding reduces quantum region
Barroca et al. [34] Generic-material demonstration	Oxygen-reduction surface reaction	Sample-based quantum diagonalization	IBM Heron R2; circuits mapped up to 80 qubits	Tractable active spaces benchmarked against classical CI	Hardware-scale demonstration; no general end-to-end speedup established
Micheletti et al. [35] Polymer demonstration	Dense lattice-polymer mixture sampling	QUBO/quantum annealing	D-Wave annealer; physical-qubit use depends on embedding	Discrete polymer statistical-mechanics formulation	Feasibility demonstrated; no universal speedup claim
Cho & Cheon [36] Composite demonstration	CFRP Cu-film placement/through-thickness conductivity	FEA-derived QUBO/quantum annealing	D-Wave Leap workflow	FEA provides design correlations and verification	Design solution demonstrated; quantum advantage not established
Cho et al. [37] Composite-design study	Natural-fibre laminate stacking/impact response	QUBO/reported quantum annealing	114 design variables; not a physical-qubit count	Classical laminate theory and Charpy impact simulation	Numerical design evaluation; experimental materials validation and general quantum advantage not established
Ishiyama et al. [55] Polymer-data + hardware demonstration	Polymer physical-property prediction	Variational QML; MERA-type circuit	IonQ ion-trap (11 available); 4-qubit model; 100-shot hardware test	Simulator comparison; hardware R^2^ rose above 0 during optimization	Limited hardware optimization; no speedup shown
Zhang et al. [56] Polymer-data demonstration	Six polymer-property predictions	PolyQT: QNN + Transformer	Computational model; best configuration: 8 qubits	Compared with classical Transformer/baselines; R^2^ = 0.27–0.92 across reported properties	Improved sparse-data prediction in study; no physical-hardware advantage demonstrated

## Data Availability

No new data were created or analyzed in this review. Data sharing is not applicable to this article.

## Abbreviations

The following abbreviations are used in this manuscript:

DFT	Density Functional Theory
MD	Molecular Dynamics
FEA	Finite Element Analysis
ML	Machine Learning
CNTs	Carbon Nanotubes
AI	Artificial Intelligence
HPC	High-Performance Computing
NISQ	Noisy Intermediate-Scale Quantum
VQE	Variational Quantum Eigensolvers
QAOA	Quantum Approximate Optimization Algorithms
QML	Quantum Machine Learning
CNOT	Controlled-NOT
QPE	Quantum Phase Estimation
XAI	Explainable Artificial Intelligence
ICME	Integrated Computational Materials Engineering
DeePMD	Deep Potential Molecular Dynamics
GAP	Gaussian Approximation Potentials
QUBO	Quadratic Unconstrained Binary Optimization
